# Differential Packaging Into Outer Membrane Vesicles Upon Oxidative Stress Reveals a General Mechanism for Cargo Selectivity

**DOI:** 10.3389/fmicb.2021.561863

**Published:** 2021-07-02

**Authors:** Nichole Orench-Rivera, Meta J. Kuehn

**Affiliations:** Department of Biochemistry, Duke University Medical Center, Durham, NC, United States

**Keywords:** extracellular vesicle, membrane vesicle, oxidative stress, enterotoxigenic *Escherichia coli*, environmental shift, membrane remodeling

## Abstract

Selective cargo packaging into bacterial extracellular vesicles has been reported and implicated in many biological processes, however, the mechanism behind the selectivity has remained largely unexplored. In this study, proteomic analysis of outer membrane (OM) and OM vesicle (OMV) fractions from enterotoxigenic *E. coli* revealed significant differences in protein abundance in the OMV and OM fractions for cultures shifted to oxidative stress conditions. Analysis of sequences of proteins preferentially packaged into OMVs showed that proteins with oxidizable residues were more packaged into OMVs in comparison with those retained in the membrane. In addition, the results indicated two distinct classes of OM-associated proteins were differentially packaged into OMVs as a function of peroxide treatment. Implementing a Bayesian hierarchical model, OM lipoproteins were determined to be preferentially exported during stress whereas integral OM proteins were preferentially retained in the cell. Selectivity was determined to be independent of transcriptional regulation of the proteins upon oxidative stress and was validated using randomly selected protein candidates from the different cargo classes. Based on these data, a hypothetical functional and mechanistic basis for cargo selectivity was tested using OmpA constructs. Our study reveals a basic mechanism for cargo selectivity into OMVs that may be useful for the engineering of OMVs for future biotechnological applications.

## Introduction

Outer membrane vesicles (OMVs) are produced when the outer membrane (OM) of Gram-negative bacteria bulges outwards producing spherical buds filled with periplasmic content and OM proteins. Bacteria ubiquitously produce these vesicles and this process is known to be genetically regulated (Bodero et al., 2007; Kitagawa et al., 2010; Kulp et al., 2015; Nevermann et al., 2019; van der Westhuizen et al., 2019). Whereas some cargo can be incorporated into vesicles by bulk flow, it has also been observed that bacteria can selectively modulate their OMV content (Bonnington and Kuehn, 2014; Schwechheimer and Kuehn, 2015; Orench-Rivera and Kuehn, 2016). However, the mechanistic details behind the preferential exclusion or selection of vesicle cargo during stress is not fully understood.

Regulated inclusion or exclusion of cargo into extracellular vesicles is considered critical to their biological roles. Enriched packaging of cargo leads to enhanced transport of particular proteins to a remote destination that can be beneficial to the cell, or can allow specific elimination of proteins that would have been toxic if they remained in the cell (Schwechheimer and Kuehn, 2013). Exclusion of particular cargo from OMVs could also be critical for the cell to preserve functionally important cellular processes. For example, our lab has previously investigated how both vesiculation levels and specific cargo selection into and exclusion from OMVs help bacteria to quickly remodel their envelope to accommodate shifts in the environment. We have shown that hypervesiculating bacteria are less sensitive to stressors than hypovesiculating mutants of *E. coli* and that vesicle production is crucial for the cell to rid itself of a detrimental accumulation of proteins in the periplasm (McBroom and Kuehn, 2007; Schwechheimer and Kuehn, 2013). Upon examination of OM LPS remodeling in *Salmonella enterica* serovar Typhimurium after a shift to and from PmrA/B-inducing conditions, it was observed that the lipid A content of OMVs significantly differed from that of the OM (Bonnington and Kuehn, 2016). The preferential release of unmodified LPS species suggested a role for OMVs to accommodate changing environmental conditions that are similar to conditions of intracellular growth within mammalian host cells. There are very few-to-no known mechanisms for OM lipids or OM-associated proteins to be quickly degraded or recycled (Schindler et al., 1980; Rassam et al., 2015), so these results are consistent with the notion that OMVs can serve as a remodeling and maintenance mechanism for the Gram-negative OM during environmental changes or stress.

Mechanisms of cargo incorporation are well-studied for eukaryotic intracellular transport vesicles (Aridor et al., 1998; Kaiser, 2000) as well as eukaryotic extracellular vesicles that derive from multivesicular bodies (Colombo et al., 2013; Li et al., 2018; Anand et al., 2019) and from the plasma membrane (Cocucci et al., 2009; Tricarico et al., 2017). However, only very few studies have addressed the mechanism behind specific cargo selection in bacteria. For instance, our previous work has shown that a mimic of a misfolded OMP intermediate recognized by the cell as a σ^E^-activating stress signal, was preferentially incorporated into OMVs, indicating OMV incorporation can be determined by the protein’s folding state, although the physical mechanism underlying the selective incorporation into the budding OMVs of cargo that contained the σ^E^-activating signal was not determined (McBroom and Kuehn, 2007). Haurat et al. (2011) revealed a mechanism in *Porphyromonas gingivalis* by which gingipains are preferentially associated with OMVs by associating with particular forms of LPS while excluding other abundant OM proteins. In *Bacteroides thetaiotaomicron*, Valguarnera et al. (2018) found that enrichment of lipoproteins in OMVs was explained by the presence of a lipoprotein export sequence that determined sorting in that system. While some studies have addressed the mechanism of cargo selection in bacteria, studies indicating environmental change-induced OMV cargo selectivity do not provide a molecular rationale. For example, Ballok et al. (2014). noted that the proteins Cif and OprF were preferentially packaged in OMVs from *Pseudomonas aeruginosa* when cells were exposed to the epoxide epibromohydrin compared to their packaging in OMVs from untreated cells, but the mechanism behind this enrichment remained elusive. Further, for *Serratia marcescens*, McMahon et al. (2012) found that, when compared to the OM, OMVs were enriched in several OM proteins (OmpA, OmpW, OmpX, MipA) when cells were grown at 30°C.

For this study we sought to investigate further aspects of OMV cargo selectivity by focusing on identifying differences in OMV protein cargo packaging in response to oxidative stress. We chose to examine a shift to oxidative conditions for several reasons. Through a high-throughput screen designed to identify *E. coli* genes involved in vesiculation, we discovered that mutations in oxidative stress response pathways were highly enriched amongst mutants with hypo- and hypervesiculation phenotypes (McBroom and Kuehn, 2007; Kulp et al., 2015) and wanted to better understand the relationship between oxidative stress and OMV production. Additionally, studies have analyzed oxidative stress induction of OMV production: van de Waterbeemd et al. (2013) found that oxidative stress caused by cysteine depletion triggered OMV release in *N. meningitidis* and Macdonald and Kuehn (2013) showed that in *P. aeruginosa*, OMV production increases after treatment with hydrogen peroxide (H_2_O_2_). Furthermore, vesicles from *H. pylori* can mitigate extracellular oxidative stress due to the packaging of the catalase KatA in the OMVs (Lekmeechai et al., 2018).

In this study we used a quantitative proteomics approach to identify the protein composition of OMVs and OM fractions, and we found significant selective packaging of different classes of proteins upon oxidative stress that was unrelated to changes in gene expression. We subsequently investigated a potential mechanism for the preferential OM retention or packaging of proteins into OMVs based on cell wall anchoring using OmpA as a model protein. Overall, this study provides insight into vesicle-mediated mechanisms of membrane remodeling in bacteria experiencing environmental shifts, as well as a general mechanistic basis for OMV protein cargo selectivity in unstressed conditions.

## Materials and Methods

### Culture Conditions

Luria Bertani (LB) broth cultures (750 mL) of enterotoxigenic *E. coli* (ETEC) strain H10407 (ATCC 35401) were inoculated with 3 mL of overnight culture and grown to stationary phase (37°C, 200 rpm, ∼16 h). Cells were pelleted (3,500 × g, 8 min, 20°C), resuspended in 750 mL fresh LB, either with or without hydrogen peroxide (20 mM), and cultures incubated for another 3 h (37°C, 200 rpm). This procedure leads to cultures considered to be in “pseudo-stationary phase” since stationary phase cells resuspended in fresh media is acknowledged to be slightly different from stationary cultures in their original spent media but is necessary to rid the media of OMVs produced in earlier phases of growth. LB broth cultures (250 mL) of BL21 constructs were inoculated with 1 mL of overnight culture and grown to stationary phase overnight at 37°C. Cells were pelleted (3,500 × g, 8 min, 20°C), resuspended in 250 mL fresh LB, either with or without hydrogen peroxide (1 mM), and cultures incubated for another 3 h (37°C, 200 rpm).

### Sytox Green Sensitivity Assay

Untreated and peroxide-treated ETEC or BL21 construct cultures were centrifuged and resuspended in 10 mM Tris-HCl buffer, pH 8. Sytox Green (Thermo Fisher Scientific) (18 μL of a 0.1 mM solution) was added to 1 mL of the bacterial suspensions and fluorescence assessed at 500 nm excitation/550 nm emission. Heat killed bacteria (100°C, 10–60 min) from untreated ETEC or BL21 cultures were used as a positive control.

### OMV Isolation

OMVs were isolated from the untreated and peroxide-treated cultures as previously described (Horstman and Kuehn, 2000). Briefly, cells were centrifuged (10,000 × g, for 10 min, 4°C) and supernatants were saved. Supernatants were filtered using 0.45 μm membrane filters (Millipore) and OMVs in the sterile supernatants were pelleted (38,400 × g, 3 h, 4°C). The OMV-containing pellets were washed by resuspending in Phosphate Buffered Saline (PBS) (10 mM dibasic phosphate, 137 mM NaCl, 2.7 mM KCl pH 8.0) and re-pelleted (91,149 × g, 1 h, 4°C).

### OM Isolation

Untreated and peroxide-treated ETEC cultures were pelleted (10,000 × g, 4°C, 10 min) and cells resuspended in PBS containing 1 mg/mL of freshly prepared lysozyme from a stock solution of 10 mg/mL in 10 mM Tris-HCl, pH 8.0. Bacteria were then lysed by French press twice at 10,000 psi, and saved for further purification.

Untreated and treated BL21 construct cultures were pelleted (4,575 × g, 20°C, 10 min) and cells resuspended in 20 mM Tris-Cl, 20% sucrose, 5 mM EDTA, pH 8.0. 100 μL of Lysozyme (15 mg/mL) was added, and the cells were incubated on ice for 40 min. After the incubation, 150 mM MgCl_2_ was added, and the cells were pelleted (4,575 × g, 20°C, 10 min). The cell pellet was then resuspended in 10 mM Tris, pH 8.0 and sonicated (7 rounds of 15 s pulses at 80% amplitude followed by 15 s of cooling). Cell fragments were pelleted by centrifugation (4,575 × g, 15 min, 4°C) and the supernatant was removed. Membranes were pelleted from the supernatant by centrifugation (40,000 × g, 1 h 4°C). The membrane pellet was washed with 10 mM Tris, pH 8.0, resuspended in dH_2_O, and membranes fractured by freezing at −80°C and thawing at room temperature. The membrane suspension was then mixed 4:5 with Sarkosyl solution (1.67% Sarkosyl and 11.1 mM Tris, pH 8.0) and incubated at room temperature for 20 min. The OM fraction was pelleted by centrifugation (40,000 × g, 90 min, 27°C), washed in 10 mM Tris, pH 8.0, and resuspended in PBS.

### OMV Quantification

For protein-based quantitation, 10 μL of each OMV sample in PBS was incubated for 10 min with 300 μL of Bradford reagent (Thermo Fisher Scientific) and then absorbance at 595 nm was measured. For lipid-based quantitation, 20 μL of each OMV sample was incubated with 20 μL of 100 μg/mL FM4-64 (Molecular Probes) and incubated at 37°C for 10 min. PBS alone was used a negative control. Protein and lipid measurements were normalized to OD_600_.

### OMV and OM Sample Purification and Preparation for Proteomics

Pelleted OMVs and OM fractions were adjusted to 45 and 36% (v/v) iodixanol (Optiprep, Greiner) by the addition of Optiprep in HS buffer (10 mM HEPES, pH 7.4; 150 mM NaCl) in a total of 0.5 mL, loaded in the bottom of a 12.5 mL ultracentrifuge tube (Beckman) and layered with decreasing percentages of OptiPrep/HS (1 mL of a 40% concentration, 1 mL 35%, 3 mL 30%, 3 mL 25%, and 2 mL 20%). Samples were centrifuged (280,755 × g, 18 h, 4°C), and fractions were removed sequentially from the top of the gradient. The iodixanol was removed by dilution in PBS followed by two rounds of high-speed centrifugation (91,149 × g, 1 h, 4°C), and the pellets were resuspended in PBS. Fractions were separated by SDS-PAGE and proteins detected using Ruby Stain. Fractions enriched in outer membrane proteins (OmpF/C and OmpA) were selected for proteomic analysis.

### Proteomic Analysis

See supplementary Methods for sample preparation, data collection and analysis performed by Duke Core proteomics facility (Supplementary Tables 1–3). For annotation of proteomic data, all protein sequences were blasted using the UniProt server (The UniProt Consortium, 2018) and matched to *E. coli* K12 strain homologs based on 90–100% homology. Subcellular localizations and information on topology and function were obtained from EcoGene (Zhou and Rudd, 2013), EcoCyc (Caspi et al., 2018), MetaCyc (Caspi et al., 2018), and PSORTb (Yu et al., 2010; Supplementary Tables 4, 5).

From the quantitative proteomic data, we determined for which proteins there were at least a twofold decrease or increase in packaging (OMV/OM ratio) for peroxide untreated (-) and treated (+) conditions (Supplementary Table 6, columns 2 and 4, respectively). We also determined which OM-associated proteins displayed a difference in packaging of at least twofold between the treated and untreated conditions, | OMV(+):OM(+) − OMV(−):OM(−)| > 1 (Supplementary Table 6, column 5) and gene ontology (GO) analysis was performed on these increased (“up”) and decreased (“down”) groups to identify significant enrichment of particular biological processes (Ashburner et al., 2000; Mi et al., 2019; Supplementary Table 7). The OM-associated proteins that changed in concentration at least twofold in the OM samples for treated (+) compared with untreated (−) conditions was also determined (Supplementary Table 6, column 6).

### Statistical Analysis and Model for Protein Sorting From Proteomic Dataset

To estimate the effect of the treatment on the protein packaging ratio (OMV:OM) and whether the effect is associated with protein type (lipoprotein vs. integral protein), we implemented a Bayesian hierarchical model. Our statistical model contains parameter β that describes how the global protein packaging ratio (y) changes with treatment (x), namely, the impact the treatment has on the log_2_ of the ratio. We also have a random effect, α, for each individual protein (accounting for the different base levels of protein expression). In Bayesian statistics we represent our prior beliefs about a parameter in terms of a probability distribution on that parameter, called a prior. After observing data, we can update our beliefs and obtain a new probability distribution, called a posterior that represents a combination of our prior beliefs and the observed data. In this case our prior for the variance of our random effect is a t-distribution and we use an improper uniform prior for β (prior does not integrate to 1 but gives a proper distribution for the posterior). While the posterior has no analytic form (a simple equation), we can draw samples from the posterior using Markov Chain Monte Carlo. From these samples we can infer properties such as mean, median, credible intervals etc., about our posterior distribution. We implement this using the brms R package (Bayesian Regression Models using Stan) (Bürkner, 2017, 2018). The exact sampling method they use to obtain these posterior samples is provided in their documentation.

The same statistical approach was used on the localization dataset to compare abundances of proteins from different cellular compartments in OMVs from treated (+) vs. untreated (−) cultures and to determine functional enrichment of periplasmic proteins (with classifiers: cell wall metabolism, chaperone, hydrolase, part of ABC transporter, redox, or other) in OMVs from treated (+) vs. untreated (−) cultures.

### Statistics for Non-proteomics Data

For direct sample size comparison, the paired *t*-test was used, and for fold comparison, the unpaired, all using Prism 8 software. The *t*-test value of ≤ 0.05 was considered statistically significant. The number of times each experiment was repeated (n) is indicated in the figure legends.

### Gene Expression Data From the GEO Database

We mined the GEO database (Edgar et al., 2002) to obtain gene expression profiles under stress for the OM proteins, lipoproteins and periplasmic proteins in our study. Gene expression 60 min after transition from anaerobic to aerobic conditions was used from the GSE4735 dataset (Partridge et al., 2006).

### Detection of Carbonyl-Modified Proteins

The Protein Carbonyl Assay Kit (ab178020, Abcam) was used to detect oxidized proteins in OMV and pelleted whole cell (4,575 × g, 20°C, 10 min) samples. Total protein was extracted from the samples from untreated and treated cultures and oxidized proteins were derived according to manufacturer instructions. Derived samples were separated using SDS-PAGE and immunoblotted using the anti-DNP antibody provided. SuperSignal West Pico PLUS Chemiluminescent Substrate (Thermo Fisher Scientific 34577) was used to detect the derivatized oxidized proteins. FIJI (ImageJ) software was used to calculate lane densities.

### qRT-PCR

Cultures of ETEC cells (25 mL) were grown overnight, cells were pelleted (4,575 × g, 20°C, 10 min), resuspended in fresh LB, either with or without hydrogen peroxide (20 mM), and cultures incubated for another 3 h (37°C, 200 rpm). After 3 h, cells were pelleted by centrifugation (4,575 × g, 20°C, 10 min) and RNA was extracted using RNeasy kit (QIAGEN) according to the manufacturer’s instructions. cDNA was synthesized with High-Capacity cDNA Reverse Transcription Kit (Applied Biosystems 4368813) and quantitative real-time PCR reactions of the listed genes were performed using SYBR Select Master Mix (Applied Biosystems 4472908) in the Applied Biosystems StepOne-plus or QuantStudio 3 Real-time PCR System, following the manufacturer’s instructions. See Supplementary Table 8 for primers synthesized (Eurofins) and used for analysis. Each sample was assayed in triplicate with wells with no cDNA used as negative control. Relative gene expression quantifications were calculated using the comparative Ct method with 16S as endogenous control using primers as described (Chakravorty et al., 2007). Fold expression changes were determined using the formula 2-ΔΔCt.

### Plasmid Constructs

His-tagged versions of YfeY, YgdI, PhoE, and TamA were synthesized with codon-optimization for expression in *E. coli* and cloned into the Multiple Cloning Site in the vector pET-23a(+)(Genscript USA Inc.). We relied on the background expression (e.g., the inherent leakiness of the T7 promoter) of the pET-23a(+) vector for expression of these constructs. OmpA constructs pGI10 (full length OmpA fused to mCherry) and pGV30 (truncated OmpA fused to mCherry) were generously provided by Dr. den Blaauwen from the University of Amsterdam (Verhoeven et al., 2013; see Supplementary Figures 8, 9 and Supplementary Table 9 for sequences and references). BL21-AI^TM^ One Shot^TM^ Chemically Competent *E. coli* (Invitrogen C607003) cells were transformed with constructs, and ampicillin was used as marker for plasmid selection. For OmpA constructs, cultures were grown to stationary phase (37°C, 200 rpm, ∼16 h) in the presence of 0.1 mM IPTG added at the start of the culture.

### Immunoblot for Validation of Protein Packaging Into OMVs

OMV and OM samples were separated using Mini-PROTEAN TGX (Tris-Glycine eXtended) Stain-Free Precast Gels (BIORAD) (for detecting YfeY, PhoE, TamA constructs) or Mini-PROTEAN Tris-Tricine Stain-Free Precast Gels (BIORAD) (for detecting YgdI constructs). Proteins were transferred onto nitrocellulose using Semidry electroblotting OWL System (Thermo Fisher Scientific) for 45 min at 200 mA. The nitrocellulose was blocked (5% milk, in TBS-T (Tris-buffered saline with 1% Tween 20), then incubated overnight with primary 6x-His Tag mouse Monoclonal Antibody (Thermo Fisher Scientific MA1-135), followed by three 5 min washes with TBS-T and a 5 min wash of TBS (TBS-T without the Tween). After incubation washes were repeated and membrane was incubated for an hour with IRDye 680RD Goat Anti-Mouse IgG (H+L) (LI-COR 925-68070) (to detect YfeY, YgdI, and PhoE) or Goat Anti-Mouse IgG H&L (Alexa Fluor 680) (ab175775, Abcam) (to detect TamA). The blot was imaged using an Odyssey Imaging System. The fluorescence (RFU) of the protein band was quantified by densitometry using FIJI and normalized using the FM4-64 measurements of the OMV and OM samples. For OmpA constructs, western blotting was performed as described using anti-RFP-HRP conjugated antibody (ab34767). After incubation washes were performed as described, and the nitrocellulose blot was incubated for 5 min with SuperSignal^TM^ West Pico PLUS Chemiluminescent Substrate (Thermo Fisher Scientific 34577). When necessary, samples were diluted so that the immunoreactive signals would be in the linear range of detection and the RFU values multiplied to account for differences in dilution. The blot was imaged using a c300 Azure biosystems Gel Imaging System. See Supplementary Figure 14 for representative blots.

## Results

### Isolation and Proteomic Analysis of OMVs Produced After Oxidative Stress

In order to investigate the effect of environmental conditions on cargo packaging into OMVs we selected a bacterial strain with a constitutively high OMV yield. We chose enterotoxigenic *E. coli* (ETEC) H10407 which our lab has previously determined produces approximately 10-fold more vesicles than non-pathogenic K-12 *E. coli* strains (Horstman and Kuehn, 2002). We also developed a treatment protocol that would cause oxidation of cellular contents, but would have negligible effects on bacterial membrane integrity and growth (Supplementary Figure 1). No significant differences in Sytox Green accessibility was detected between cells isolated from treated cultures and untreated cultures when stationary phase ETEC cultures incubated for 3 h at 37°C in the presence or absence of up to 20 mM hydrogen peroxide, revealing intact membranes (Supplementary Figure 2). We note that this concentration of hydrogen peroxide is higher than typically used in reports of oxidative stress in laboratory (K12) strains of *E. coli*, and that higher concentrations (up to 50 mM) of hydrogen peroxide are usually necessary to induce stress in studies of pathogenic *E. coli* (Bearson et al., 2009; Felicio et al., 2009; Łoś et al., 2010; Mei et al., 2017). To validate that our treatment conditions nevertheless did result in oxidation of ETEC proteins, we analyzed whole cell extracts and observed an increase in oxidative carbonyl modifications (Supplementary Figure 3). Since the 3 h shift to media containing 20 mM hydrogen peroxide caused cellular protein to be oxidized but did not perturb membrane integrity, we used these conditions in our further analyses. Unlike some other species examined in previous studies (Macdonald and Kuehn, 2013; van de Waterbeemd et al., 2013), a comparison of OMV yield by protein and lipid assays in treated and untreated culture supernatants revealed that the oxidative treatment did not significantly alter OMV production by ETEC (Supplementary Figure 4). These data, albeit preliminary, demonstrate that for ETEC a non-lethal shift with peroxide leads to the generation of oxidative species but not OMV induction.

We next used proteomic analyses to investigate whether ETEC OMV cargo changed upon oxidative stress. In order to evaluate OMV packaging, the ratio of cargo proteins in the OMVs compared with the OM, it was critical to isolate and compare OMVs and OM from the same stationary phase culture. Stationary phase cultures of ETEC were centrifuged, the media replaced with fresh LB to exclude OMVs produced during earlier growth phases, and the cultures incubated for 3 h at 37°C during which time they generated OMVs. The OM fraction of the cells and OMV preparations from the culture supernatants were then isolated and gradient-purified from three independent replicate cultures for quantitative proteomic mass spectroscopy (MS) analysis.

Proteins with at least two peptides present in the OM and OMV proteomic datasets were selected for further analysis and annotated (Supplementary Figure 5). Proteins in the preparations were categorized as extracellular (including pili, phage and flagellar), OM, periplasmic, inner membrane (IM), cytoplasmic, and unknown (if no information about localization was found in the databases) according to annotations individually cross-referenced using literature and database searches (Supplementary Tables 4, 5). Relative abundance within the preparations was determined based on the quantitative MS intensities, and, as expected, the data revealed that a majority (90.07%) of the proteins in the OMV preparation were designated to be located in the OM, periplasm, or extracellular milieu (Supplementary Figure 6). The sensitivity of the technique also yielded proteins from other subcellular locations, and while it is not known whether those are or are not specifically part of OMVs, previously published proteomic analyses of OMVs yielded similar results (Lee et al., 2007; Choi et al., 2011). For further analyses we focused on OM-associated proteins, since this class would be present in both the OMV and OM preparations, unlike soluble (periplasmic) proteins which we did not quantitatively assess for cargo packaging in this study.

### Statistical Model Reveals Differential Sorting of Lipoproteins and Integral Proteins During Stress Conditions

To evaluate OMV cargo selectivity, the average intensity for each protein in the OMV and OM samples was calculated and a ratio determined based on the peptide abundance. We first determined the amount differentially packaged cargo from OMVs produced by ETEC grown in unshifted culture conditions. For these pseudo-stationary phase ETEC cultures, eleven OM-associated proteins were either enriched or excluded at least twofold as cargo in OMVs compared to their abundance in the cellular OM (highlighted in yellow and blue in Supplementary Table 6, columns 1 and 3). These data revealed a basal level of OMV cargo selectivity for stationary phase ETEC in unstressed conditions.

Oxidative treatment of the ETEC cultures yielded significantly different cargo packaging results. Specifically, a cursory inspection of the types of OM-associated proteins in the differentially packaged groups suggested a substantial difference between the localization of OM lipoproteins and integral OM proteins (Supplementary Table 6). OM lipoproteins appeared to be selectively exported (14/44, highlighted in gray in Supplementary Table 6, column 4) with fewer being selectively retained (9/44, highlighted in red in Supplementary Table 6, column 2), whereas integral OM proteins appeared to be selectively retained upon stress (20/44, highlighted in green in Supplementary Table 6, column 2) and none exhibited selective OMV packaging (0/44). When we further interrogated the dataset regarding whether the cargo exhibited a > 2-fold change in packaging upon oxidative stress, we found that a substantial majority (23/32, highlighted in blue in Supplementary Table 6, column 5) of the OM-associated cargo that decreased in the amount packaged into OMVs with stress, were integral OM proteins. Conversely, a substantial majority (19/21, highlighted in orange in Supplementary Table 6, column 5) of the OM-associated cargo that increased in the amount packaged into OMVs with stress were OM lipoproteins. These data suggested a stress-induced selectivity of OMV cargo based on the topological class of the OM-associated protein.

**FIGURE 1:**
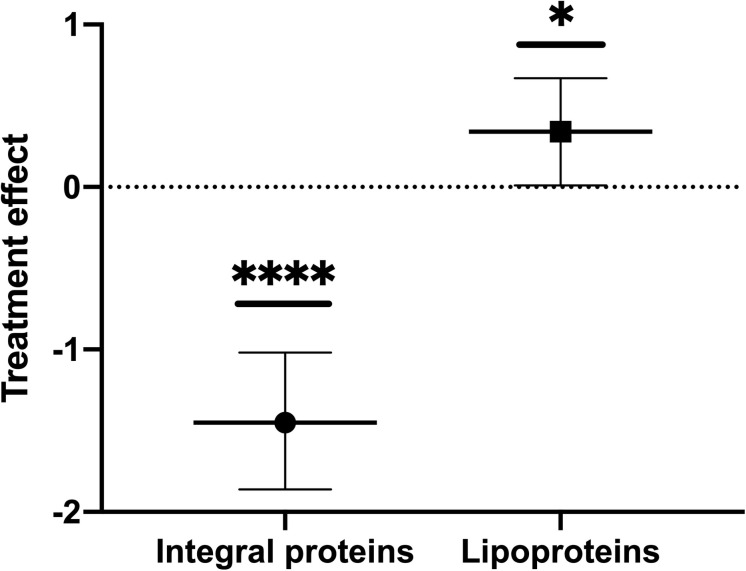
Oxidative stress causes a preferential export of lipoproteins into OMVs and a preferential retention of integral proteins in the OM of ETEC. Parameter values for the treatment effect on OMV packaging ratios were calculated for OMV(+):OM(+), and OMV(–):OM(–) to obtain the increase or decrease in protein packaging in OMVs due to treatment stress. Error bars are confidence intervals. *P*-values calculated from confidence intervals (treatment effect): ^∗^*p* ≤ 0.05, ^****^p ≤ 0.0001.

Since our dataset was only based on a triplicate sample size and thus with high variance in average intensities, not atypical for proteomic data, we were unable to evaluate the results statistically for individual proteins. Instead, we examined the data by applying Bayesian Hierarchical linear modeling, specifically suited for analysis of data with small *n*, high variance, and containing natural groupings (in this case, the proteins were topologically either OM lipoproteins or integral OM proteins). The method also accounts for hierarchy or nested data. We applied this model and observed that the packaging of the two groups, OM lipoproteins and OM integral proteins, indeed changed upon treatment. The results indicated that OM lipoproteins were significantly preferentially packaged (with treatment decreasing the fold packaging value and a *p*-value of ≤0.05) in comparison to OM integral proteins (with fold values increased as a result of treatment and a *p*-value of ≤0.0001) (Figure 1 and Supplementary Figure 16). Thus, the initial observation of differential packaging based on the average values calculated for each protein was confirmed by the hierarchical model analysis of the data.

**FIGURE 2:**
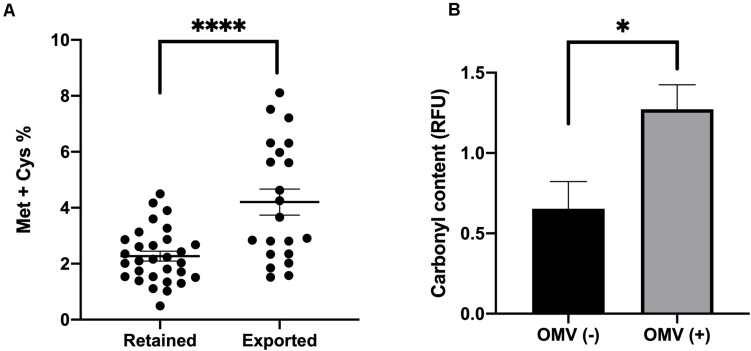
Levels of oxidizable residues and oxidized proteins are increased in OMV cargo in response to oxidative stress. **(A)** Methionine and Cysteine percentage (Met and Cys residues per total amino acids in protein without initial Met) in preferentially exported vs. preferentially retained proteins in oxidized samples. **(B)** Densitometric Oxyblot analysis of OMV proteins from 3 h H_2_O_2_ treated (+) or untreated (–) ETEC cultures using FIJI software and normalized to non-derivatized samples (*n* = 6). Error bars are SEM. *P*-values according to the Student’s *t*-test: ^∗^*p* ≤ 0.05, ^****^p ≤ 0.0001.

As our prior research indicated that misfolded protein is selectively packaged into OMVs (Schwechheimer and Kuehn, 2013), we also wondered whether proteins that are more prone to be oxidized due to stress are more likely to be exported in OMVs after a shift to hydrogen peroxide-containing media. We mined the UNIPROT database for proteins that were either preferentially exported in OMVs or retained in the OM in our analysis and calculated percentage of methionine and cysteine residues in each protein (disregarding the initial Met). The results revealed that, compared to retained proteins, exported proteins had significantly more Met or Cys residues (Figure 2A). We also observed an increase in carbonyl modifications present in our OMV samples from treated cultures compared to untreated cultures (Figure 2B and Supplementary Figure 7). Together these data revealed that, in addition to lipoproteins, proteins more prone to oxidation are more likely to be packaged in OMVs.

### No Correlation Is Observed Between Packaging of Proteins Into OMVs and Gene Expression Under Stress Conditions

**FIGURE 3:**
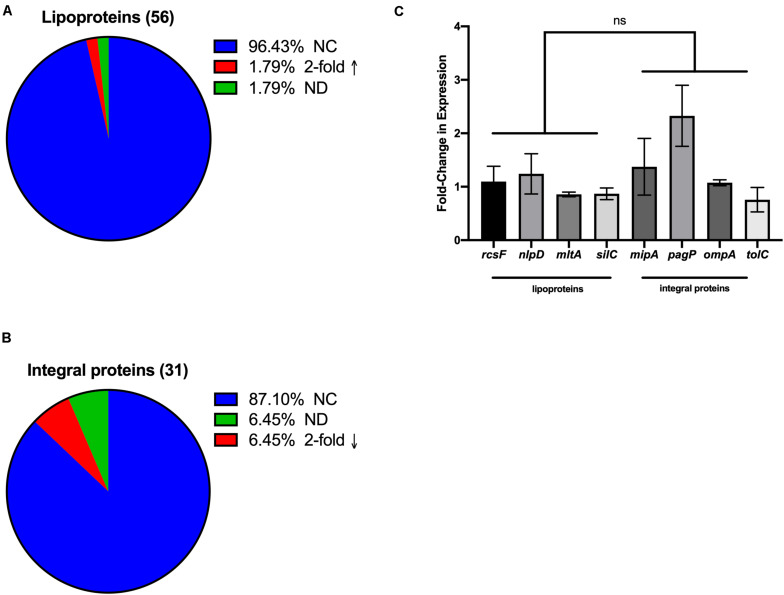
Selective OMV packaging is not a direct reflection of expression levels for the different classes of OMV cargo. **(A,B)** Changes of a twofold increase or decrease from database mining of GEO profile GSE4735 for gene expression after shift from anaerobic to aerobic conditions. NC, no change; 2-fold ↑, = at least twofold increased expression; 2-fold ↓, = at least twofold decreased expression; ND, not in database. **(C)** Fold change in expression was measured by qRT-PCR after 3 h H_2_O_2_ treatment compared to untreated controls for randomly selected genes encoding OM lipoproteins or integral OM proteins in ETEC. Error bars are SEM; ns, not significant.

Because it is known that increases of envelope proteins incorporated into vesicles can occur by overexpression of those proteins (McBroom and Kuehn, 2007), we wondered whether the observed preferential incorporation of OM lipoproteins compared with integral proteins upon oxidative stress was due to oxidation-induced differences in expression of the sets of genes encoding these proteins. To address this question, we mined the GEO profiles dataset of the NCBI (Edgar et al., 2002; Barrett et al., 2013), specifically GSE4735 (Partridge et al., 2006). This dataset reports on the dynamics of oxidative stress-induced gene expression and was useful for our analysis since the transition from anaerobic to aerobic conditions has been noted to be comparable to oxidative stress (Cabiscol et al., 2000). The gene expression profiles for OM integral proteins and lipoproteins in this dataset were classified by their gene expression profiles according to their packaging into OMVs. Only 2% of the OM lipoproteins identified in our study showed increased gene expression under the stress conditions shown in the GEO dataset using a twofold threshold (Figure 3A). As for the OM integral proteins, only 6.4% showed decreased expression (Figure 3B). Although this analysis is based on data using other *E. coli* strains, it is relevant since genetic regulatory elements such as the response to oxidative stress are typically well-conserved within a bacterial species (with ETEC having an 88% symmetrical identity with *E. coli* str K-12 substr. MG1655; Crossman et al., 2010). Therefore, it appeared that the selective packaging of OM lipoproteins over integral proteins into OMVs that we observed for hydrogen peroxide stressed ETEC is likely not simply due to an oxidation-induced increase in their gene expression.

In order to validate that levels of gene expression were not the direct cause of oxidation-induced cargo selection in our ETEC strain, four lipoprotein genes and four integral protein genes were chosen at random out of our OMV cargo datasets. We isolated RNA from unshifted and oxidatively stressed ETEC cultures and used qRT-PCR to evaluate whether increases or decreases in gene expression directly correlated with packaging patterns of these classes of proteins. Our results showed that lipoproteins were not expressed more than integral OM proteins after a shift to oxidative stress (Figure 3C), supporting the conclusion that there is not a correlation between the observed selective packaging of different classes of proteins and gene expression.

### Validation of Differential OM-Associated Protein Class Sorting Upon Oxidative Stress

To test our hypothesis that differential export and retention of classes of OM-associated proteins occurs as a result of oxidative stress, we quantified the incorporation into OMVs of OM lipoprotein and integral OM protein cargo in a standard laboratory *E. coli* strain. Lipoproteins YfeY and YgdI and integral proteins PhoE and TamA were chosen for evaluation as OMV cargo since they had previously been tagged with His_6_ at their C-terminus without interfering with their localization or stability (Van Gelder et al., 1996; Shen et al., 2014). Non-pathogenic *E. coli* strain BL21 cells were transformed with expression plasmids encoding the tagged proteins (Supplementary Figures 8, 9) and the constructs expressed without using a transcriptional inducer. The expected OM localization of all four tagged proteins was confirmed by immunoblotting of subcellular fractions (Supplementary Figure 10). Oxidative stress conditions were also optimized for the BL21 strains. A lower concentration of peroxide (1 mM) was used compared with the conditions used for ETEC cultures, given the lower tolerance to oxidative stress in non-pathogenic strains (Supplementary Figure 11). Membrane integrity was confirmed to be uncompromised for all the BL21 constructs treated with 1 mM H_2_O_2_ (Supplementary Figure 12).

**FIGURE 4:**
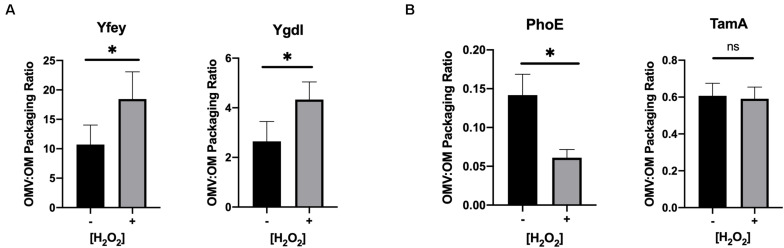
Validation of preferential packaging of lipoproteins into OMVs upon oxidative stress. Packaging ratios (OMV:OM) were calculated from the detection of tagged OM lipoproteins **(A)** or integral OM proteins **(B)** by quantitative immunoblotting of OM and OMV preparations from untreated (–) and 3 h H_2_O_2_-treated (+) BL21 constructs. YfeY (*n* = 8), YgdI (*n* = 4), PhoE (*n* = 6), TamA (*n* = 6). Error bars are SEM. *P*-values according to the Student’s *t*-test: ^∗^*p* ≤ 0.05.

OMVs and OM from untreated and hydrogen peroxide-treated uninduced cultures of the transformed constructs were purified as described earlier for the ETEC preparations. The OMV:OM packaging ratio for each cargo protein was calculated based on quantitative immunoblotting of purified OMV and OM preparations from untreated and treated cultures. Upon oxidative treatment, OM lipoproteins YfeY and YgdI both showed an increase in packaging into OMVs, and integral OM protein PhoE showed a decrease in OMV packaging (Figure 4). The integral OM protein TamA did not show a significant difference in packaging. The results observed for 3 out of 4 of these randomly selected proteins are consistent with our hypothesis that generally, but not in all cases, oxidative stress leads to differential OMV packaging of OM lipoprotein and integral OM proteins in *E. coli*.

### Physical Tethering to the Cell Wall Determines Packaging of Proteins Into OMVs

We considered how the distinctive general functions and features of lipoproteins and integral OM proteins could govern cargo selectivity. Gene Ontology analysis revealed that, amongst all the OM proteins we detected, the proteins that were at least twofold more retained upon oxidative stress were enriched for those related to transport and iron homeostasis (see Table 1 and Supplementary Table 7). In contrast, those proteins that showed increased packaging into OMVs upon stress revealed no significant enrichment in any GO biological process category (Table 1). One characteristic in common for this set of proteins is their interaction with cell envelope components such as peptidoglycan (PG) and/or IM components such as the TonB/ExbBD and AcrAB systems (Zgurskaya and Nikaido, 1999; Husain et al., 2004; Pawelek et al., 2006; Shultis et al., 2006). This discovery suggested a mechanistic basis for cargo retention: protein connections with other cell envelope components reduce entry into OMVs, and oxidative stress exacerbates such mechanistic differences in cargo availability.

**TABLE 1:** Preferentially retained OM-associated proteins are enriched in transport and iron homeostasis GO categories.

GO biological process	Preferentially retained proteins		Preferentially exported proteins	
	Fold enrichment	*P*-value	Fold enrichment	*P*-value
Siderophore transmembrane transport	90.9	3.52E-10	<0.01	1.00E00
• Iron ion transmembrane transport	70.7	3.83E-11	<0.01	1.00E00
• Siderophore transport	60.6	3.41E-12	<0.01	1.00E00
Gram-negative-bacterium-type cell outer membrane assembly	45.6	3.36E-06	<0.01	1.00E00
Bacteriocin transport	45.4	6.95E-05	<0.01	1.00E00
Protein localization to membrane	21.6	4.69E-04	11.0	9.08E-02
Iron ion homeostasis	19.4	7.70E-06	<0.01	1.00E00
Lipid transport	18.9	8.63E-06	<0.01	1.00E00

In order to test our mechanistic hypothesis of cargo selectivity, we used two versions of the integral protein OmpA to model envelope-tethered and untethered OM proteins for several reasons. OmpA is one of the most abundant OM proteins in *E. coli* with an eight-stranded beta barrel N-terminal domain and a soluble C-terminal domain located in the periplasm (Ortiz-Suarez et al., 2016) which can non-covalently bind to PG (Park, 2011). OmpA has also been previously employed to isolate OMVs (Alves et al., 2017) and to direct loading into OMVs through protein fusions (Alves et al., 2015, 2016a,b) due to its abundance in the OM and OMVs. Notably, both the epitope-tagged constructs of full-length OmpA (encoded by pGI10) and truncated OmpA lacking the PG-binding domain (encoded by pGV30) have been previously determined to be stable and folded properly in the membrane (Verhoeven et al., 2013). We confirmed that this was also the case in our assay conditions (Supplementary Figure 13). Critically, these features allowed us to avoid issues of membrane and cargo instability commonly complicating the accurate evaluation of packaging of non-native OMV cargo independent of folded state. Further, in *S. enterica* the periplasmic domain of OmpA has also been implicated in the modulation of the open/closed states of OmpA during oxidative stress to regulate membrane permeability (van der Heijden et al., 2016), thus its behavior upon oxidative stress is of physiological interest.

**FIGURE 5:**
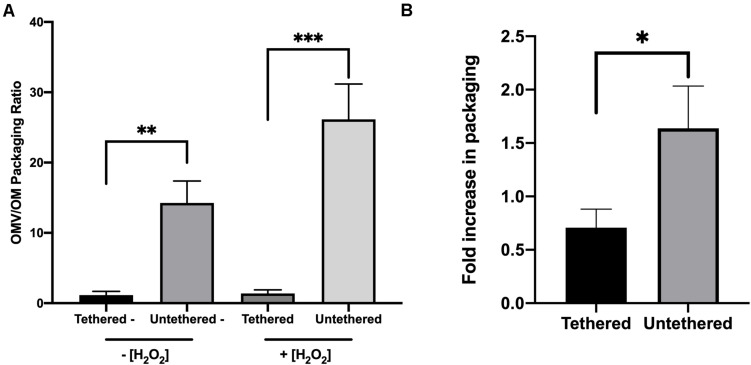
Selective packaging of truncated OmpA compared to full-length OmpA into OMVs is exacerbated with oxidative stress. Packaging ratios (OMV:OM) for full-length OmpA and truncated OmpA were determined by immunoblotting for untreated BL21/pGI10 and BL21/pGV30 cultures, respectively, for unstressed cultures and stressed cultures **(A)**. **(B)** OMV:OM packaging ratios for the full-length and truncated OmpA constructs in unstressed cultures from part A was compared to that for 3 h H_2_O_2_-treated cultures and fold-change calculated (*n* = 6 for pGI10, *n* = 5 for pGV30). Error bars are SEM. *P*-values according to the Student’s *t*-test: ^∗^*p* ≤ 0.05, ^∗∗^*p* ≤ 0.01, ^∗∗∗^*p* ≤ 0.001.

We first tested whether protein interaction with the cell wall was a potential mechanism for OM protein sorting into OMVs. The data showed a significant preferential packaging of the truncated version of OmpA into OMVs in comparison with the full-length version of the protein (Figure 5A). Using treatment conditions verified to maintain membrane integrity (Supplementary Figure 15), we further noted a significant increase truncated OmpA selectivity into OMVs under oxidative stress (Figure 5B). These data suggest that protein packaging into OMVs is controlled by physical tethering of the proteins to cell envelope components and that oxidative stress exacerbates this effect.

## Discussion

By quantitatively comparing the protein composition of *E. coli* OMVs and cellular OM produced during unstressed and oxidatively stressed conditions, this study revealed the unanticipated differential sorting of two classes of OM embedded proteins into the secreted OMVs. The data informed a mechanistic hypothesis of OM imbedded cargo selectivity into vesicles based on differences in cell wall tethering that was validated using model cargo protein constructs.

Previous studies have shown that upon stress, bacteria either increase OMV production, or increase the selectivity of damaged or immature cargo into OMVs, or both (Orench-Rivera and Kuehn, 2016). In this study, we did not observe a significant increase in ETEC OMV production after oxidative stress. It is possible that any benefit mediated by increased levels of OMV production during oxidative conditions, for example to remove damaged or misfolded envelope proteins, are already met by the high constitutive level of OMV production by ETEC. Indeed, we observed an increase in oxidative stress-induced modifications to OMV proteins. While this was not surprising, since vesicles were derived from oxidized cultures, a closer look at the composition of the proteins preferentially exported (after calculating difference of at least twofold between treated and untreated samples) in comparison to the ones preferentially retained in the membrane, revealed a higher amount of oxidizable resides. These data show that proteins more likely to have been oxidized were enriched for export upon stress. The enrichment of proteins with oxidizable residues is consistent with a model in which OMVs help rid the cell of oxidatively modified proteins in response to oxidative stress. However, simply packaging damaged protein did not appear to explain the proteomic analysis regarding the observed differential packaging of classes of OM protein.

After annotating and examining differences in retained and exported ratios of the various polypeptides, we were able to detect trends in the selective packaging of specific classes of OM-localized proteins. Based on a low sample size (*n* = 3) and high variance in the content for the OM and OMV preparations, not uncommon for bacterial subcellular fractionation (Baik et al., 2004; Ebanks et al., 2005; Sezonov et al., 2007; Liu et al., 2008; Nikaido, 2009; Sridhar and Steele-Mortimer, 2016), statistically significant differences were not found for the differential packaging of individual proteins in treated or untreated conditions. Instead, we implemented a statistical model to analyze the changes in fold values of the two protein classes found in our study: lipoproteins and integral proteins (Efron et al., 2001; Gottardo et al., 2006; Manda et al., 2007; Walker et al., 2008). The results showed a significant preferential export of OM lipoproteins in comparison to integral OM proteins.

We considered several explanations for the observed differential packaging of the two classes of OM cargo. First, our experimental methodology could have resulted in the biased enrichment of different classes of proteins in the preparations. However, we note that by using lysozyme and French press methods to obtain OM for analysis, we avoided the issue that detergent can reduce the abundance of lipoproteins in OM samples (Zhang et al., 2018). We also analyzed whether the sorting trends we observed were due to corresponding changes in gene expression, but found no direct correlation between the differential incorporation of lipoproteins and integral proteins and gene expression during oxidative stress conditions in *E. coli* databases. Expression results for several randomly selected gene candidates also did not reveal any correlation between packaging patterns and gene expression of these protein classes. We further wondered if the selectivity we observed was related to the observed enrichment of lipoprotein cargo into OMVs discovered in a study of *B. thetaiotaomicron* (*Bt*) (Valguarnera et al., 2018). In addition to the fact that *Bt* contains surface-exposed lipoproteins, a feature not found in *E.* coli, the lipoprotein sorting mechanism is further distinct from that found in this study. Notably, the preferential OMV packaging of lipoproteins in *Bt* depends on a canonical signal sequence that is not found in the ETEC lipoproteins. Further, lipoprotein sorting into OMVs was not compared for unstressed and stressed cultures of *Bt*, thus it is not known if the selectivity is environmentally regulated. Therefore, the differential cargo sorting into OMVs that we observed appears distinct and mediated by a previously unknown mechanism.

**FIGURE 6:**
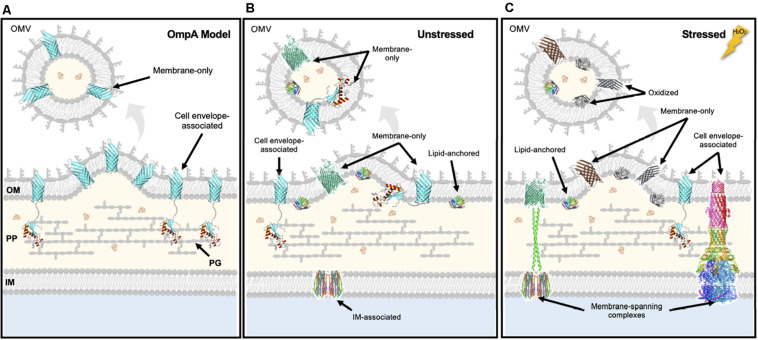
Summary for the selective OMV packaging of untethered cargo into OMVs in unstressed and stressed conditions. **(A)** Results from studying the packaging of OmpA constructs revealed that cell envelope-associated (full-length) cargo tend to be retained whereas membrane only (truncated) cargo tend to enter OMVs. Results from studying selective native cargo OMV packaging during unstressed **(B)** and stressed **(C)** conditions revealed that the ability of proteins to tether to the cell envelope is a determinant for incorporation as OMV cargo. Integral OM proteins engaged in cell envelope complexes which increase cellular efflux and nutrient import are preferentially retained in the membrane after stress, maybe whereas oxidized proteins as well as untethered OM proteins will be preferentially exported into OMVs. OM, outer membrane; IM, inner membrane; PP, periplasm; PG, peptidoglycan. OmpA structure from Ishida et al. (2014); AcrAB-TolC by Wang et al. (2017); LolB (lipoprotein), PDB 1IWM; TonB extensions modeled from PDB 1JCD; ExbB/ExbD from PDB 5ZFV; FhuA from PDB 2GRX; OmpX from PDB 1QJ8.

The observed significant preferential retention of OM integral proteins over lipoproteins under oxidized conditions led us to hypothesize that distinct protein topology within the envelope might be a general determinant for retention or export. Specifically, most OM lipoproteins that were unlikely to be engaged with other envelope components were significantly enriched in the exported fraction whereas the integral OM proteins discovered to be more significantly retained in the cells in our study were mostly iron transporters that are known to associate with cell wall and IM components (Table 1 and Supplementary Tables 6, 7). By using the two OmpA constructs that differ in their ability to bind the cell wall, we were able to test this theory. The data showed that truncated version of OmpA was significantly more packaged than the full-length version under unstressed conditions (Figure 6A). We acknowledge the limitations of this evaluation, as we grossly simplified the OM cargo into two topological classifications, “tethered vs. untethered,” to model the difference between OM lipoproteins and integral OM proteins. Nevertheless, these data support a mechanism wherein OMV budding events occur within sites of low membrane anchoring, causing proteins in these sites to be incorporated as OMV cargo, whereas integral proteins that are engaged with components of the envelope are retained (Figure 6B). Our experimental results also support an exclusively proteomic study of *Neisseria meningitidis* OMVs demonstrating that the constitutively-produced vesicles have a relatively low abundance of membrane-anchoring proteins compared to the OM (Lappann et al., 2013). For some OM-associated proteins, there are changes in tethering during stress, and thus this selective export/retention mechanism can be exacerbated upon environmental changes (Figure 6C).

Envelope structure has been found to impact levels of OMV production, and the current study contributes to this model that changes in envelope structure also affect OMV cargo packaging. Gram-negative bacteria can modulate vesiculation by altering PG-OM cross-linking via localized modulation of PG degradation and synthesis (Schwechheimer and Kuehn, 2015). Accumulation of PG or LPS fragments led to an increase in vesiculation by pushing the OM outwards causing cargo to be incorporated in sites where the OM had decreased levels of cell wall-bound Lpp (Braun’s lipoprotein) (Schwechheimer et al., 2014). Whether these effects are coordinated with cargo selectivity remains to be explored.

There is likely to be a direct, evolved relationship between the functional cellular benefits and the envelope tethering-dependent differential packaging classes of OM-associated components into OMVs. As mentioned earlier, OMVs benefit cells as a stress response by enabling the elimination of toxic, damaged envelope components (McBroom and Kuehn, 2007; Schwechheimer and Kuehn, 2013), and this was further corroborated by findings here that proteins containing residues more susceptible to oxidative damage material were the enriched in the OMVs. However, we considered whether the cell also benefited from the inverse situation—increased retention of cargo important to the cell with reduced levels of export via OMVs. Several results support this potentially functional benefit of cargo selectivity. We found the packaging differential between the full-length and truncated OmpA further increased upon stress, and we note that OmpA plays a role in oxidative response and that beneficial role coincides with PG association (van der Heijden et al., 2016). In addition, of the 23 integral OM proteins whose exclusion as OMV cargo increased after oxidative stress, nearly all were also measured in higher abundance in the OM after the stress (highlighted in blue, Supplementary Table 6, columns 5 and 6). Therefore, the cell preferentially retained the subclass of OM proteins that are transporters and these are important in nutrient acquisition and export which may be needed to maintain cellular homeostasis during or after oxidative stress. The cargo that shifted to become more released as OMV cargo upon oxidative stress were mainly OM lipoproteins that also became less abundant in the OM after the stress (15/21, highlighted in orange, Supplementary Table 6, columns 5 and 6). Whether increased OM lipoprotein content would be harmful to the cells following oxidative stress, or whether this shift in OM abundance was a repercussion of spatial constraints from having an increased retention of the integral OM proteins remains unclear. In sum, the results support our model that selective cargo retention *vs*. release in OMVs resulting from differences in envelope tethering can generate changes in the OM composition which could be beneficial to the cell, particularly in response to an environmental stress.

Sudden stresses, such as changes in environmental conditions are a common occurrence for bacteria. ETEC, for instance, is known to colonize the surface of fruits and vegetables and is present in environmental water; however, once in a mammalian host, it is suddenly exposed to acidic and alkaline shifts in the gastrointestinal tract, shifts in carbon sources, and attack by competing bacteria as well as the host immune response (Gonzales-Siles and Sjöling, 2016). The outermost barrier against stress is the cell envelope, and during changing conditions bacteria are forced to not only metabolically adapt, but also change this barrier to survive. While transcriptional responses play a role, we now add that the cell envelope connectivity can contribute to the ability of bacteria to adjust to drastic or rapid changes in the environment by influencing cellular retention and OMV-mediated protein export. Insight into general biological principles gained in studies like this can likely be useful in optimizing the engineering of biologically active nanoparticles and membrane vesicles and OMV-based vaccines for a variety of applications pertinent to ecology and human health.

## Data Availability Statement

The datasets presented in this study can be found in online repositories. The names of the repository/repositories and accession number(s) can be found below: Duke Express Repository, https://discovery.genome.duke.edu/express/projects/upload/4458.

## Author Contributions

NO-R and MK conceived and designed this study and its experiments. NO-R conducted experiments and drafted the manuscript. MK helped to draft, edit, and finalize the manuscript. Both authors contributed to the article and approved the submitted version.

## Conflict of Interest

The authors declare that the research was conducted in the absence of any commercial or financial relationships that could be construed as a potential conflict of interest.
