# Supplementary material for: Differential Packaging Into Outer Membrane Vesicles Upon Oxidative Stress Reveals a General Mechanism for Cargo Selectivity
Source: Front Microbiol. 2021 Jul 2;12:561863. doi: 10.3389/fmicb.2021.561863 (PMC8284480; doi:10.3389/fmicb.2021.561863)
Supplement: Supplementary file 1 [file Data_Sheet_1.pdf]

Supplementary Material

1 Supplementary Figures

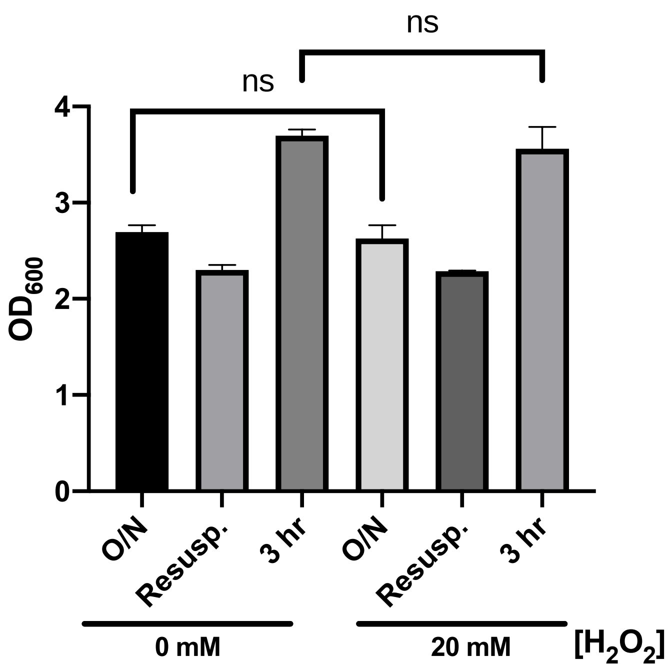

Supplementary Figure 1. Shift conditions do not compromise bacterial cell growth. OD_{600} of ETEC cultures was assayed after overnight growth (O/N), after resuspension in fresh media (Resusp.) and after 3 hr incubation (3 hr) with 0 mM or 20 mM H_2O_2. Error bars are SEM. ns, not significant (n=3).

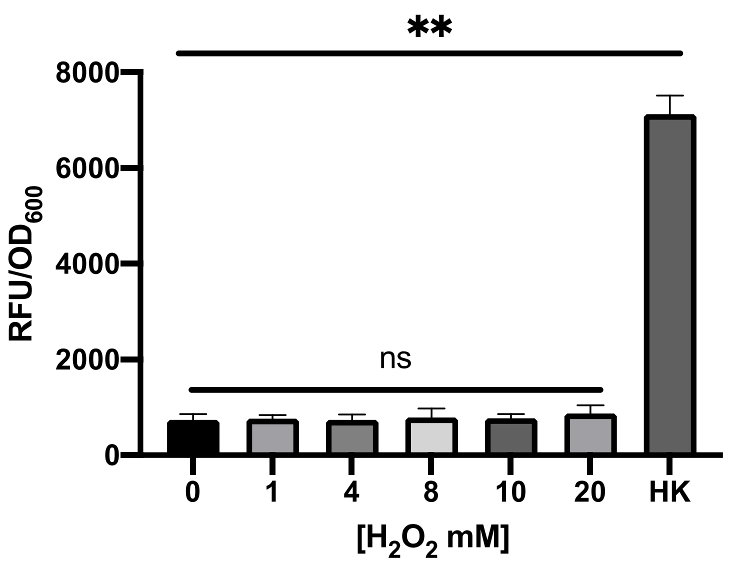

Supplementary Figure 2. Shift conditions do not compromise membrane integrity. Relative fluorescence (RFU) normalized by OD_{600} of ETEC cultures assayed with Sytox Green at 3 h after treatment with the indicated H_2O_2 concentrations. HK, heat-killed cell control. Error bars are SEM. P-values according to the Student's t test: ** $p \leq 0.01$, ns, not significant (n=3).

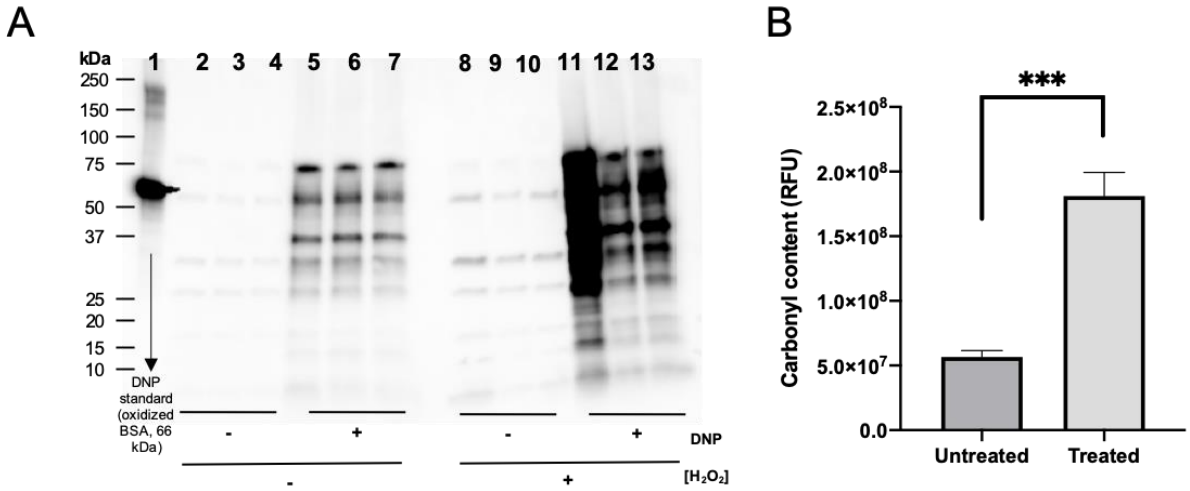

Supplementary Figure 3. H_2O_2 treatment oxidizes protein in ETEC. (A) Representative Oxyblot analysis of triplicate whole cells preparations from ETEC cultures treated for 3h with H_2O_2 (+) or mock treated (-). Derivatization with DNP is indicated (+) and underivatized samples are included as controls (-). DNP-derivatized oxidized bovine serum albumin (BSA) is included as a control in Lane 1. (B) Densitometric measurements of the Oxyblots of whole cell preparations from untreated and peroxide-treated cultures using FIJI software. Error bars are SEM. P-values according to the Student's t test: *** $p \leq 0.001$ (n=6). The migration of molecular weight standards are indicated (kDa).

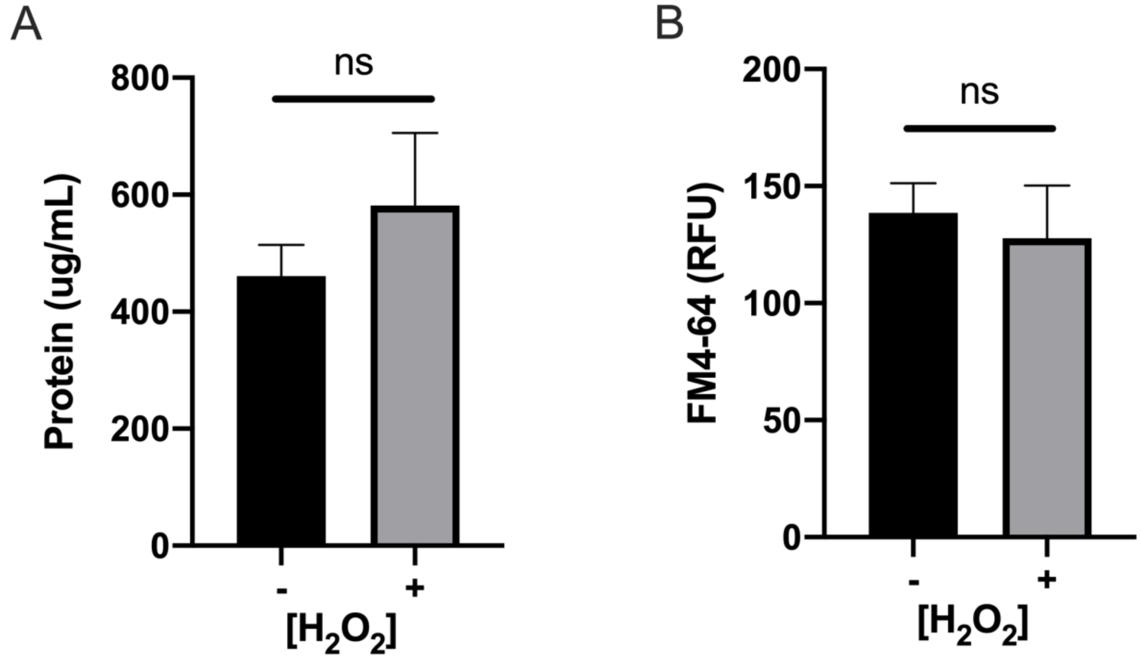

Supplementary Figure 4. Oxidative stress does not alter vesicle production in ETEC. OMV production measured by Bradford (protein content) **(A)** and FM4-64 incorporation (lipid content) **(B)** after a 3 h treatment with 20 mM H_2O_2 (+) or a mock shift (-) Values were normalized to the OD_{600} of the cultures at time of OMV harvest. (n=6). Error bars are SEM. ns, not significant.

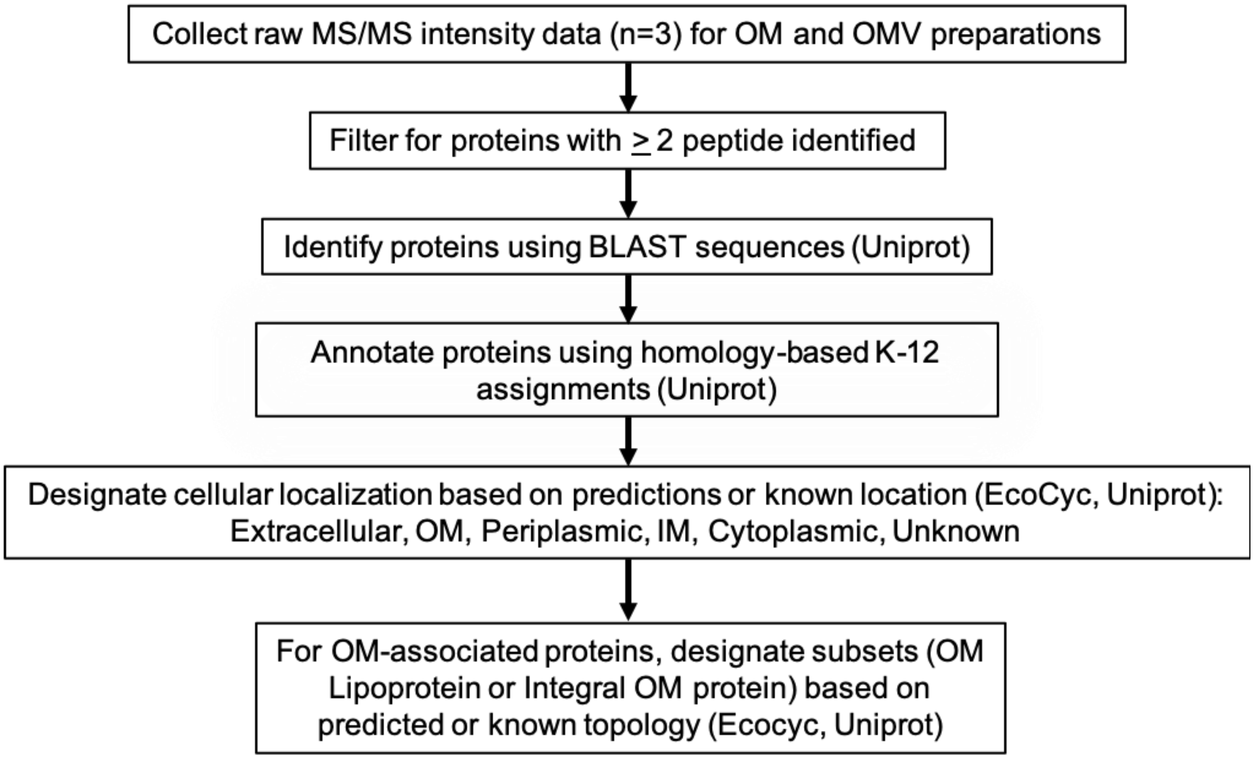

Supplementary Figure 5. Proteomic data identification and annotation process. Databases used listed in the Materials and Methods section.

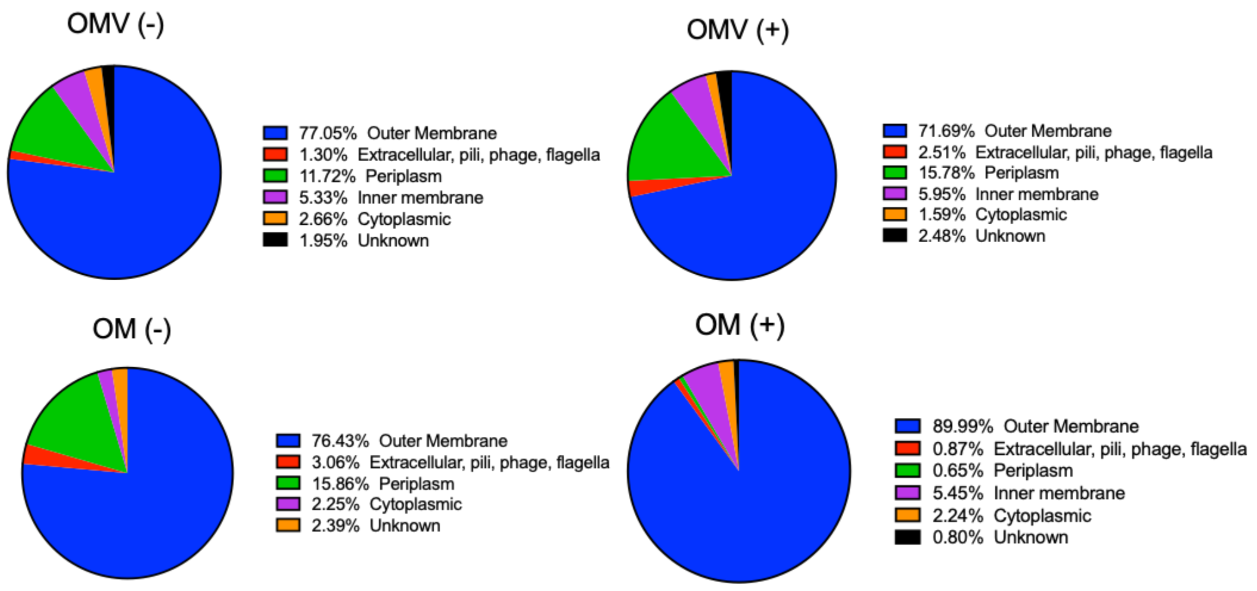

Supplementary Figure 6. Subcellular localizations of proteins in OMV and OM preparations from untreated (-) and treated (+) cultures. The percentage of proteins annotated for each compartment was calculated from the average individual peptide intensities for those proteins which had 2 or more peptides identified by proteomics. Localization data obtained from UniProt and pSortB.

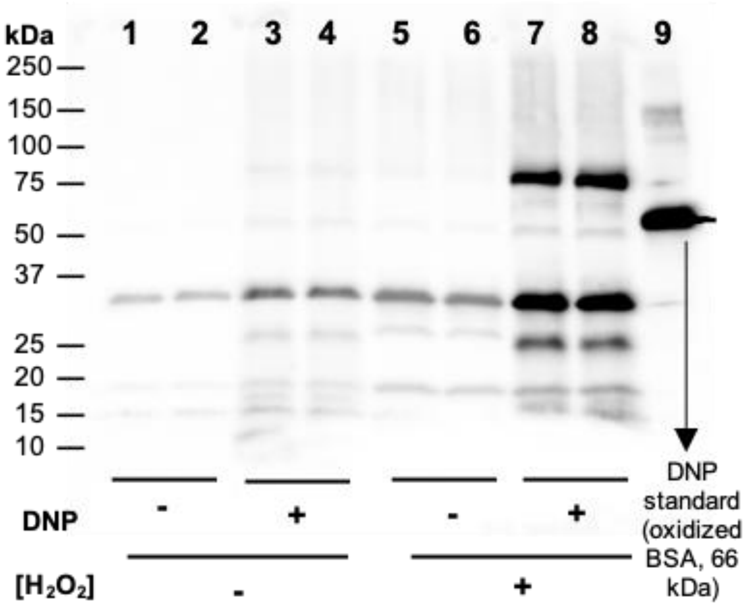

Supplementary Figure 7. OMV cargo is more oxidatively modified in response to stress. Representative Oxyblot of duplicate OMV samples from ETEC cultures treated (+) or untreated (-) with H_2O_2. Derivatization with DNP is indicated (+) and underivatized samples are included as controls (-). DNP-derivatized oxidized bovine serum albumin (BSA) is included as a control in Lane 9. The migration of molecular weight standards are indicated (kDa).

YfeY

Missing from 3D structure model:

STMSSVNWSAANPWNWFGSS

Vector: pET23a(+), in frame
N-terminal Signal sequence and acylation site:
MKSLRLMLCAMPLMLTGC
C-terminal Thrombin cleavage site (LVPR/GS)
followed by His_6 tag

```
P76537  TKVSEQGVGELTASTPLQEQAIADALDGDYRLRSGMKTANGNVVRFFEVMKGDNVAMVIN  98
2qzb.A  TKVSEQGVGELTASTPLQEQAIADALDGDYRLRSGMKTANGNVVRFFEVMKGDNVAMVIN  73

P76537  GDQGTISRIDVLDSDIPADTGVKIGTPFSDLYSKAFGNCQKADGDDNRAVECKAEGSQHI  158
2qzb.A  G--GTISRIDVLDSDIPADTGVKIGTPFSDLYSKAFGNCQKA------AVECKAEGSQHI  133

P76537  SYQFSGEWRGPEGLMPSDDTLKNWKVSKIIWRR                             191
 2qzb.ASYQFSGEWRGPEGLMPSDDTLKNWKVSKIIWRR                             166
```

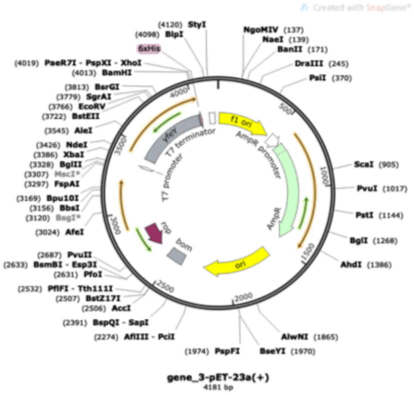

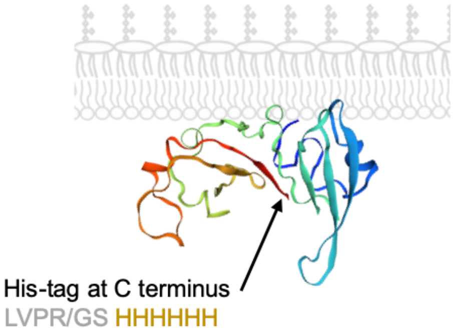

YgdI
Vector: pET23a(+), in frame
N-terminal Signal sequence and acylation site:
MKKTAAIISACMLTFALSAC
C-terminal Thrombin cleavage site (LVPR/GS)
followed by His_6 tag

```
    MODEL SGSNYVMHTNDGRTIVSDGKPQTDNDTGMISYKDANGNKQQINRTDVKEMVELD 74
2ra2.2.ASGPNYVMHTNDGRSIVTDGKPQTDNDTGMISYKDANGNKQQINRTDVKEMVALE 55
```

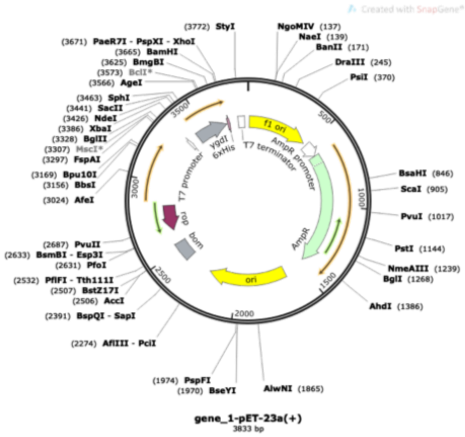

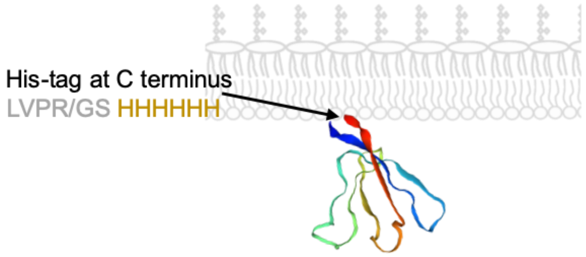

Supplementary Figure 8. Lipoprotein constructs used in this study. Features of the construct encoding the C-terminally His_6-tagged lipoproteins are summarized as well as a graphical model of their 3-dimensional structure relative to a membrane, color coded from blue to red, N- to C-terminus, respectively (Bienert et al., 2017).

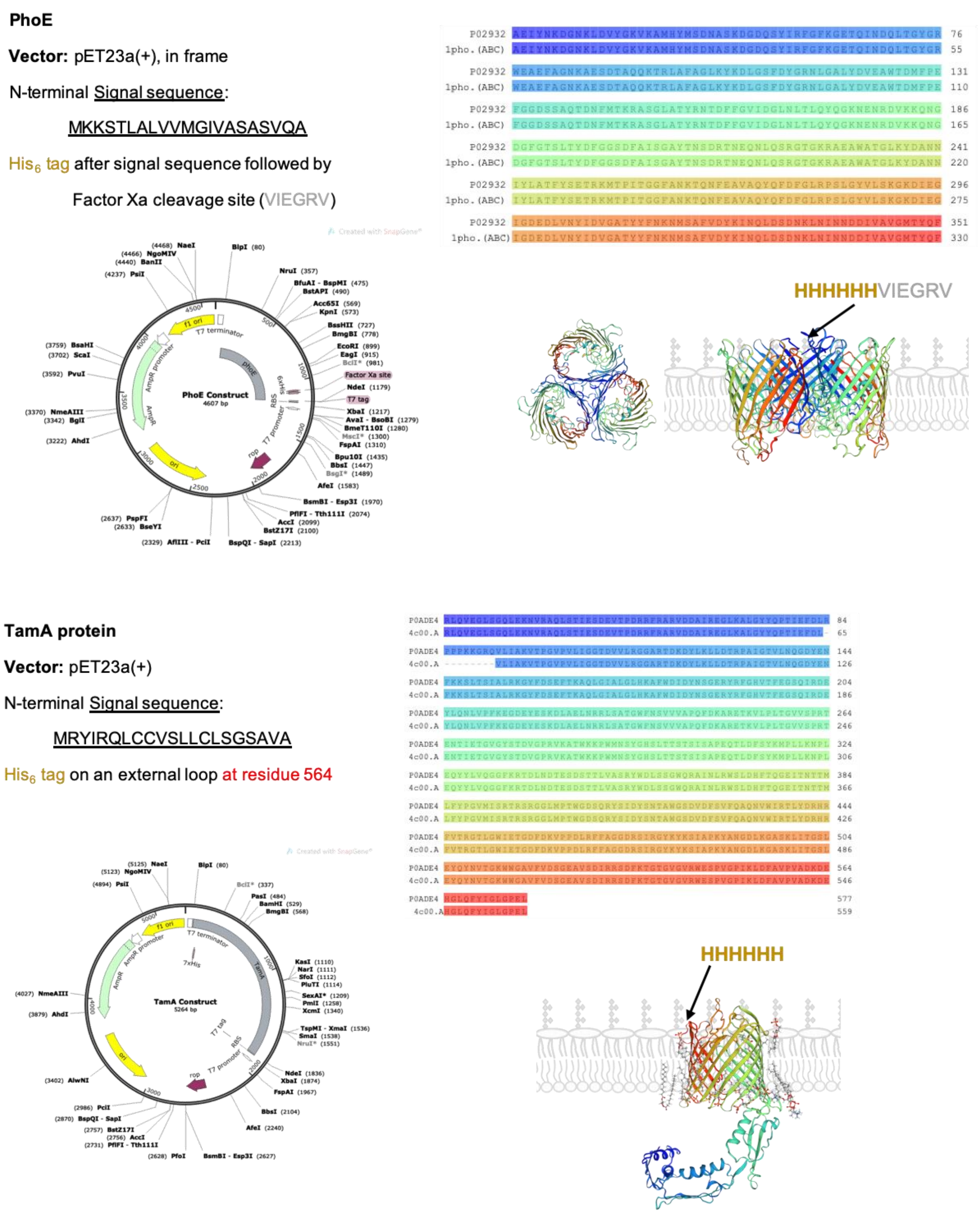

Supplementary Figure 9. Integral protein constructs used in this study. Features of the construct encoding the His_6-tagged OM proteins are summarized as well as a graphical model of their 3-dimensional structure relative to a membrane, color coded from blue to red, N- to C-terminus, respectively (Bienert et al., 2017).

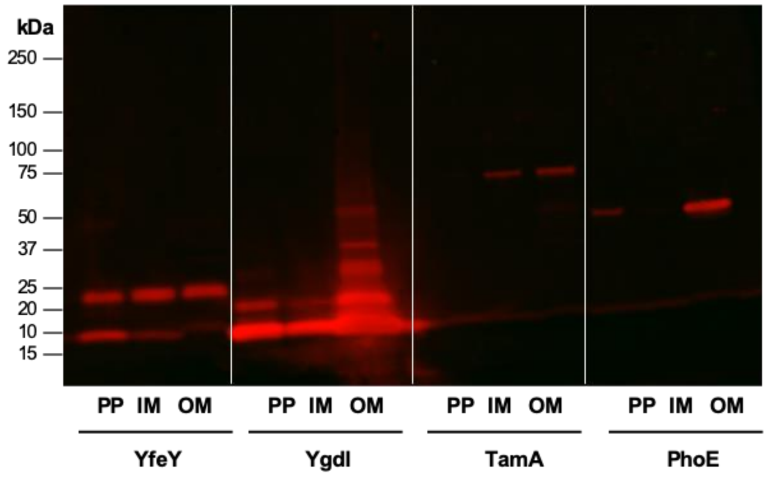

Supplementary Figure 10. Outer membrane localization of tagged constructs. Subcellular fractions (equal concentrations of protein) were separated by SDS-PAGE and detected by immunoblotting. PP, periplasm; IM, inner membrane; OM, outer membrane. The migration of molecular weight standards are indicated (kDa).

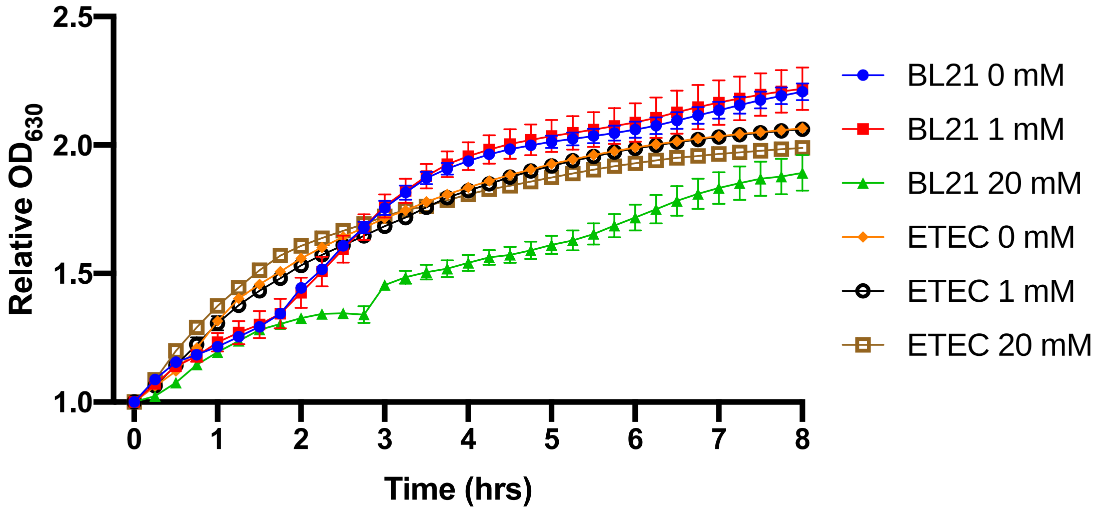

Supplementary Figure 11. *E. coli* strain BL21 is more sensitive to H_2O_2 than ETEC. Cells were inoculated onto 96-well plate from a 5 mL overnight culture at a dilution of 1:5000 in media and the indicated concentration of H_2O_2. Cultures were incubated for 8 h at 37°C, and OD_{630} was recorded every 15 min. OD_{630} values were normalized to starting OD_{630} values at time 0.

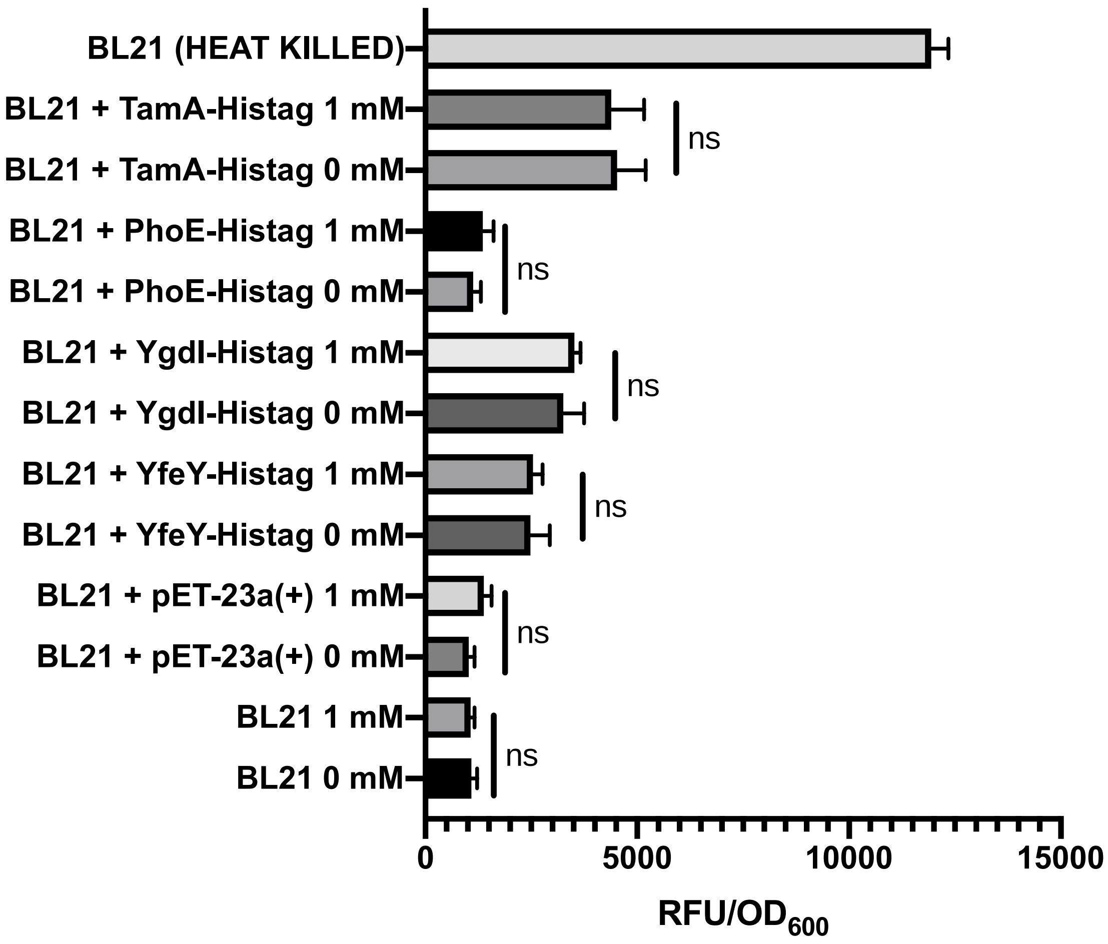

Supplementary Figure 12. Oxidative stress does not compromise membrane integrity relative to untreated cultures. Relative fluorescence (RFU) normalized to OD_{600} of the indicated BL21 construct cultures assayed with Sytox Green at 3h after treatment with 1 mM H_2O_2 or mock treated. Heat-killed cell control is shown. (n=4). Error bars are SEM; ns, not significant.

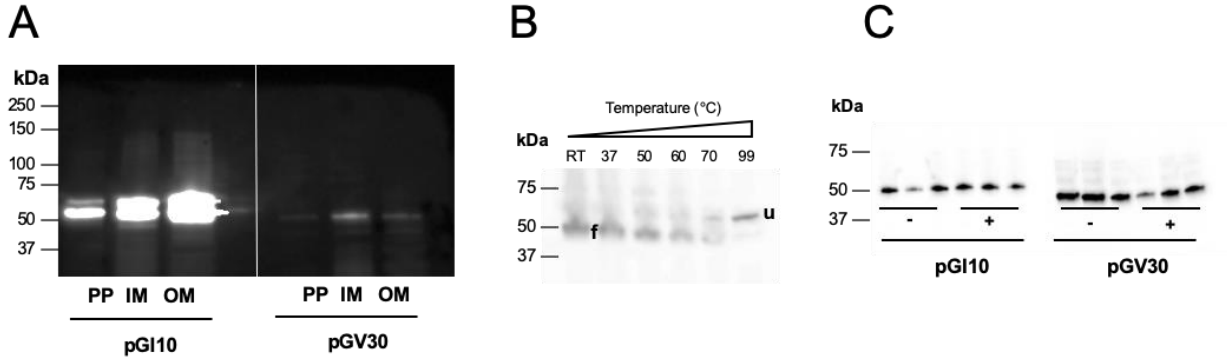

Supplementary Figure 13. Proper localization and folding of tagged OmpA constructs in the OM and OMVs. (A) Subcellular fractions (equal concentrations of protein) were separated by SDS-PAGE and detected by immunoblotting. PP, periplasm; IM, inner membrane; OM, outer membrane. **(B)** pGV30 OM fractions were resuspended in 5X SDS-PAGE sample buffer and either not heated (RT), or heated at 37°C for 5 min, at 50°C, 60°C and 70°C for 15 min, or at 99°C for 10 min. and detected by immunoblotting. Folded and unfolded versions of the protein are indicated by f- and u- next to the bands, respectively. **(C)** OMVs purified from the indicated mock-treated (-) and 1 mM H_2O_2-treated (+) BL21 cultures expressing the OmpA constructs were analyzed in triplicate by SDS-PAGE without boiling and detected by immunoblotting. The location of the OmpA construct bands at ~50 kDa indicates they were in a folded state in the OMVs. The migration of molecular weight standards are indicated (kDa).

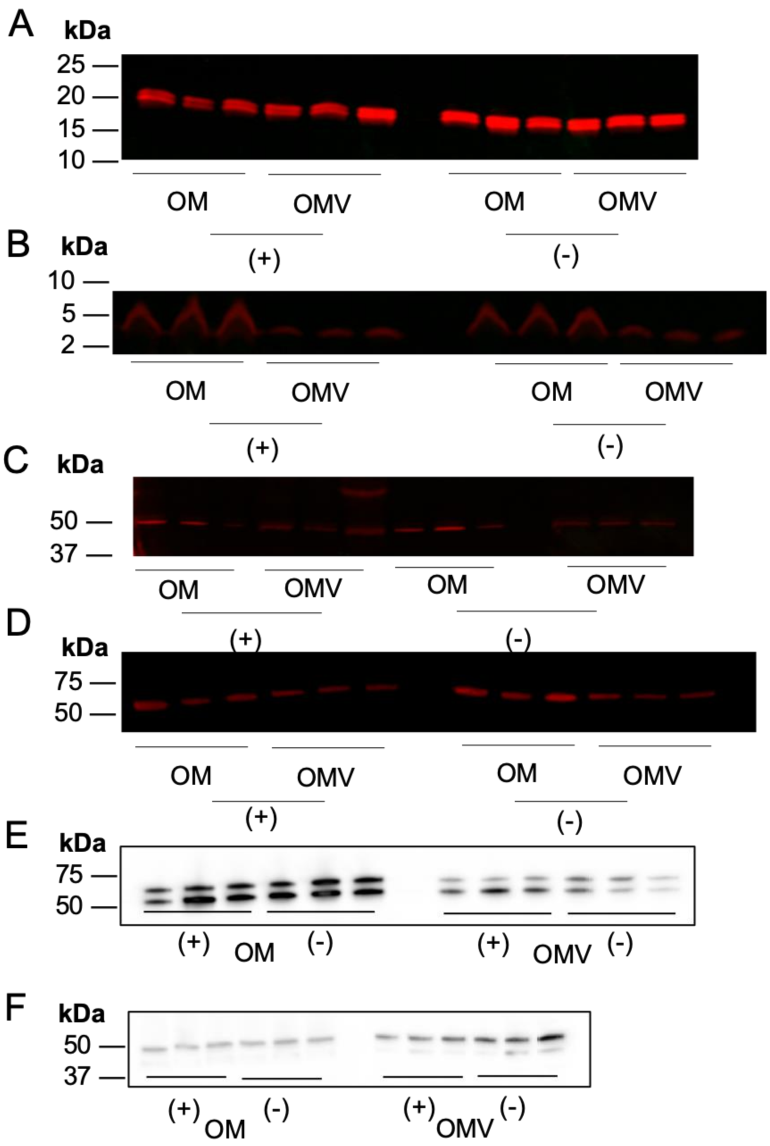

Supplementary Figure 14. Representative blot for OMV packaging of YfeY (A), YgdI (B), PhoE (C), TamA (D), pGI10 (E), and pGV30 (F). in the absence (-), 0 mM or presence (+) of 1 mM of H_2O_2.

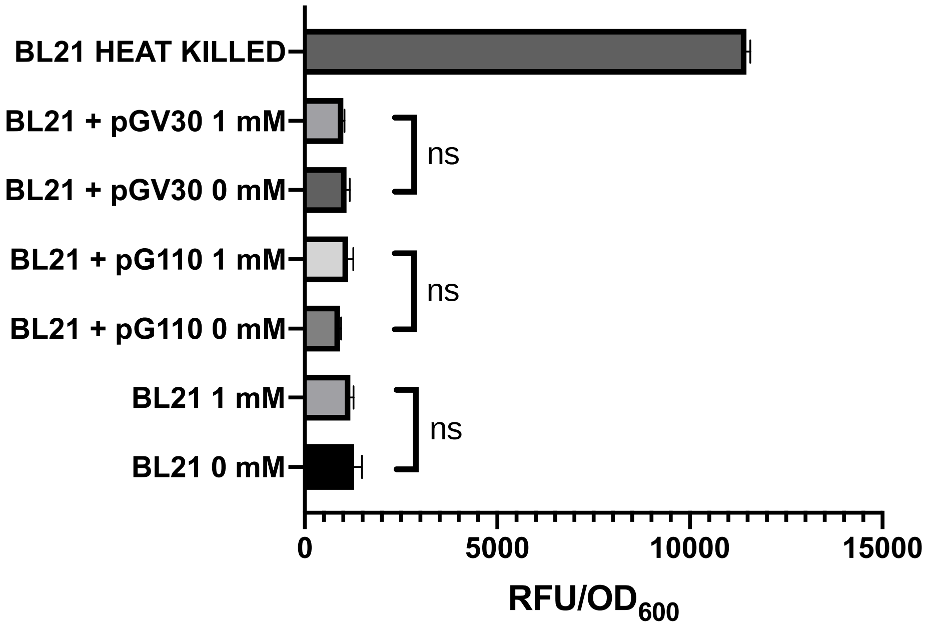

Supplementary Figure 15. Shift conditions do not compromise membrane integrity relative to heat killed control and untreated samples. Relative fluorescence (RFU) normalized to OD_{600} of the indicated BL21 construct cultures assayed with Sytox Green at 3h after treatment with 1 mM H_2O_2 or mock treated. Heat-killed cell control is shown. (n=4). Error bars are SEM; ns, not significant.

A

Integral proteins

Group-Level Effects

	Estimate	Est. Error	l-95% CI	u-95% CI	Eff. Sample	Rhat
sd (Intercept)	0.24	0.16	0.01	0.60	1398	1.00

Population-Level Effects

Intercept	0.03	0.16	-0.29	0.34	4000	1.00
Treatment	-1.45	0.22	-1.86	-1.02	4000	1.00

Family Specific Parameters

sigma	1.53	0.08	1.38	1.69	4000	1.00

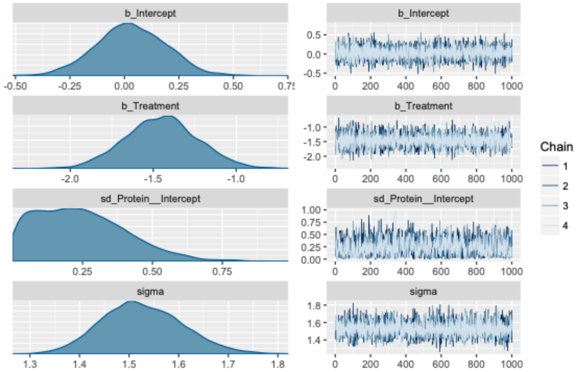

B

Lipoproteins

Group-Level Effects

	Estimate	Est. Error	l-95% CI	u-95% CI	Eff. Sample	Rhat
sd (Intercept)	0.12	0.09	0.00	0.33	2313	1.00

Population-Level Effects

Intercept	-0.16	0.12	0.00	0.33	4000	1.00
Treatment	0.34	0.17	0.01	0.67	4000	1.00

Family Specific Parameters

sigma	1.60	0.06	1.49	1.72	4000	1.00

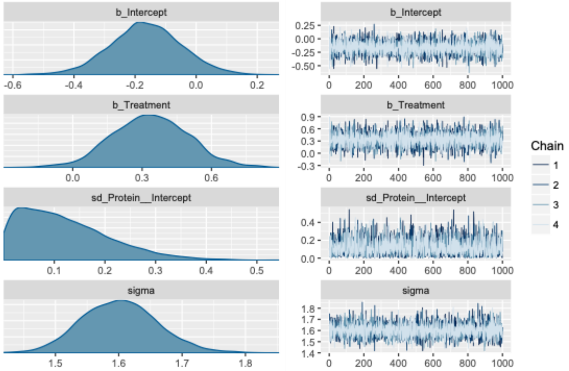

Supplementary Figure 16. Bayesian estimates and posterior distributions for the effect treatment has on integral OM (A) and OM lipoprotein (B) packaging into OMVs.

2 Supplementary Methods

2.1 Growth curves

Cells were inoculated onto 96-well plate from a 5 mL overnight culture diluted 1:5000. Cultures were incubated for 8 hours at 37°C in the presence or absence of H_2O_2 (0, 1 and 20 mM) and OD_{630} was monitored and recorded every 15 minutes using a plate reader.

2.2 Quantitative Mass Spectrometry

Membranes were solubilized by probe sonication in 50 µl of 0.2% acid labile surfactant (ALS-1) in 50 mM ammonium bicarbonate, pH 8 (AmBic). After centrifugation, solubilized proteins were quantified in supernatants by Bradford assay. Five micrograms of each sample (except for "OM(-)$_3$", which had a low yield) were normalized to equal volumes with solubilization buffer, DTT was added to a final concertation of 10 mM, and samples were reduced and denatured by heating at 80 ºC for 10 min. Next, samples were alkylated with 20 mM iodoacetamide in the dark for 30 min. Sequencing grade modified trypsin (Promega; 1:50 w/w trypsin:protein) was added, and proteins were digested at 37 ºC overnight. Samples were then acidified by addition of a final concentration of 1% (v/v) trifluoroacetic acid (TFA) and 2% (v/v) acetonitrile (MeCN), followed by heating at 60 ºC for 2 h, to inactivate trypsin and degrade ALS-1. Trypsinized yeast alcohol dehydrogenase 1 (MassPrep, Waters) at 50 fmol per µg as an internal standard following by centrifugation. Supernatants were transferred to Maximum Recovery LC vials (Waters). QC pools were prepared by mixing equal volumes of all samples.

Quantitative one-dimensional liquid chromatography, tandem mass spectrometry (1D-LC-MS/MS) was performed on 250 ng of the peptide digests per sample in singlicate, with additional analyses of conditioning runs and QC pools, as described in Table S1. Samples were analyzed using a nanoACQUITY UPLC system (Waters) coupled to a QExactive Plus high-resolution accurate mass tandem mass spectrometer (Thermo) via a nanoelectrospray ionization source. Briefly the sample was first trapped on a Symmetry C18 180 µm × 20 mm trapping column (5 µl/min at 99.9/0.1 v/v H2O/MeCN) followed by an analytical separation using a 1.7 µm Acquity HSS T3 C18 75 µm × 250 mm column (Waters) with a 90 min gradient of 5 to 40% MeCN with 0.1% formic acid at a flow rate of 400 nl/min and column temperature of 55 °C. Data collection on the QExactive Plus MS was performed in data-dependent acquisition (DDA) mode

with a 70,000 resolution (@ m/z 200) full MS scan from m/z 375 to 1600 with a target AGC value of 1e6 ions followed by 10 MS/MS scans at 17,500 resolution (@ m/z 200) at a target AGC value of 5e4 ions. A 20 s dynamic exclusion was employed. The total analysis cycle time for each sample injection was approximately 2h. Following 17 total UPLC-MS/MS analyses (including 2 conditioning runs and 3 replicate QC injections, data was imported into Rosetta Elucidator v.4 (Rosetta Biosoftware, Inc.), and analyses were separately aligned based on the accurate mass and retention time of detected ions ("features") using PeakTeller algorithm in Elucidator. Relative peptide abundance was calculated based on area-under-the-curve (AUC) of the selected ion chromatograms of the aligned features across all runs. The MS/MS data was searched against a custom Swissprot/Trembl database with E. coli O78:H11 taxonomy (downloaded on 07/18/16) with additional proteins, including yeast ADH1 and bovine serum albumin, as well as an equal

number of reversed-sequence “decoys” for false discovery rate determination (9,650 total entries). Mascot. Distiller and Mascot Server (v 2.5, Matrix Sciences) were utilized to produce fragment ion spectra and to perform the database searches. Database search parameters included fixed modification on Cys (carbamidomethyl) and variable modifications on Asn and Gln (deamidation). An additional database search was performed using semitrypsin specificity on unmatched MS/MS spectra. After individual peptide scoring using the PeptideProphet algorithm in Elucidator, the data was annotated at a 0.9% peptide false discovery rate. For quantitative processing, the data was first curated to contain only high-quality peptides with appropriate chromatographic peak shape and the dataset was intensity scaled to the robust mean.

Expression values for total of 9,740 peptides and 1,468 proteins were exported to Excel (Tables S2 and S3). Note that the expression value of each protein represents the aggregate of peptide expression values for that particular protein (Reidel et al., 2011). Of these, 1,022 proteins were quantified by 2 or more peptides, a criterion for higher confidence of identification and quantification. In order to assess technical reproducibility, we calculated %coefficient of variation (%CV) for each protein across the three injections of a QC pool that were interspersed throughout the study. The mean %CV of the QC pools was 9.7% for all proteins and 6.0% for proteins quantified by 2 or more peptides.

3 References

Bienert, S., Waterhouse, A., De Beer, T.a.P., Tauriello, G., Studer, G., Bordoli, L., and Schwede, T. (2017). The SWISS-MODEL Repository-new features and functionality. *Nucleic acids research* 45**,** D313-D319.

Reidel, B., Thompson, J.W., Farsiu, S., Moseley, M.A., Skiba, N.P., and Arshavsky, V.Y. (2011). Proteomic profiling of a layered tissue reveals unique glycolytic specializations of photoreceptor cells. *Mol Cell Proteomics* 10**,** M110.002469.
